# A Review of Possible EEG Markers of Abstraction, Attentiveness, and Memorisation in Cyber-Physical Systems for Special Education

**DOI:** 10.3389/frobt.2021.715962

**Published:** 2021-08-31

**Authors:** Maya Dimitrova, Hiroaki Wagatsuma, Aleksandar Krastev, Eleni Vrochidou, J. David Nunez-Gonzalez

**Affiliations:** ^1^ Department of Interactive Robotics and Control Systems, Institute of Robotics, Bulgarian Academy of Sciences, Sofia, Bulgaria; ^2^ Department of Human Intelligence Systems, Graduate School of Life Science and Systems Engineering, Kyushu Institute of Technology (KYUTECH), Kitakyushu, Japan; ^3^ Human-Machines Interaction (HUMAIN) Lab, Department of Computer Science, International Hellenic University (IHU), Kavala, Greece; ^4^ Engineering School of Gipuzkoa—Eibar Section, Department of Applied Mathematics, University of Basque Country (UPV/EHU), Bilbao, Spain

**Keywords:** EEG marker, abstraction, novelty, surprise, involuntary attention, memory, curiosity, cyber-physical systems for special education

## Abstract

Cyber-physical systems (CPSs) for special education rely on effective mental and brain processing during the lesson, performed with the assistance of humanoid robots. The improved diagnostic ability of the CPS is a prerogative of the system for efficient technological support of the pedagogical process. The article focuses on the available knowledge of possible EEG markers of abstraction, attentiveness, and memorisation (in some cases combined with eye tracking) related to predicting effective mental and brain processing during the lesson. The role of processing abstraction is emphasised as the learning mechanism, which is given priority over the other mechanisms by the cognitive system. The main markers in focus are P1, N170, Novelty P3, RewP, N400, and P600. The description of the effects is accompanied by the analysis of some implications for the design of novel educational scenarios in inclusive classes.

## Introduction

A process of radical transformation of present-day schools is taking place, forcefully accelerated by the recent pandemic. On the one hand, the transformation process is based on the “inclusive class” paradigm, where all children share a common learning “ecosystem” of merging ordinary and special education methods. On the other hand, technology is mediating the process and posing additional demands on the cognitive exchange of ideas, knowledge, attention focus, etc., during classroom learning. Within this ongoing, seemingly chaotic transformation of the nature of the school, the question remains: What is the most essential characteristic of a school, defining it as a necessary and sufficient condition? On a very abstract level, it can be said that a school, by definition, is a “teacher and a bunch of kids”. All other accessories of the teaching process, such as textbooks, laptops, blended learning environments, are supplementary to the main figures “the teacher and one or more learners”. The main role of the teacher is to convey new knowledge to the learner as education is a socially mediated process (e.g., Lieberman, 2007). And, when the human teacher is assisted by a humanoid robot, education will be more efficient than today, provided the robot’s activities resemble the roles and functions of the educator, personally conveying, in a social way, new knowledge to the learner, according to the learner’s individual style and cognitive needs (e.g. Dimitrova and Wagatsuma, 2015; Dimitrova et al., 2020). Detecting and diagnosing mental states of the learners, however, is a challenge to the overall approach yet a realistic aim (e.g., Schartner et al., 2015).

It is essential to substantiate the main claims for expected cognitive advantage for the learner with recent neuro-cognitive studies, while demonstrating the influence of the new technology on the learner well-being. In this, two important aspects are outlined—the human–teacher resemblance^1^ (HTD) of the robot and the aspect of special education (SED) in the classroom setting of the inclusive class. The analysed EEG and eye-tracking experiments support theories that help in drawing conclusions, which are relevant to the inclusion of both dimensions in the learning scenarios.

## Learning Processes Revealed by Observing EEG Activations

Several important brain activation effects are described in the present article within a theoretical approach of analysing the ability of the human brain to build probability distributions of the subjective influences of stimulus events as in Lindskog et al. (2021). This theory has a long-term tradition within psychometrics—more specifically, in unidimensional and multidimensional scaling (see Thurstone, 1927; Torgerson, 1958)—yet relatively new in the analysis of the underlying neuronal activity of mental states. More specifically, “Thurstone’s model assumes that an option’s quality is a Gaussian random variable” (Tsukida and Gupta, 2011, p. 3), where “option” is understood as any stimulus, possessing a “quality”, being assessed by the human. The “quality” is the dimension of the subjective effect, exerted on the subject by the perceived stimulus quality, for example, the colour of an object, the pitch of a sound, the duration of an event, impression of a painting, difficulty of a task, etc. The figures below are in line with the mathematical assumptions of these models and represent probability distributions of psychological effects, resulting from the discussed underlying brain mechanisms. Typically, the form of this probability distribution function is “bell” shaped, that is, a normal distribution of the stimulus effect on the cognitive system is assumed with all formal attributes—mean and standard deviation tailored to the specific stimulus effect (McLeod, 2019). The actual scale values are determined empirically or experimentally (Torgerson, 1958).

### P1—Response to Abstraction and the Role of Involuntary Attention

**FIGURE 1:**
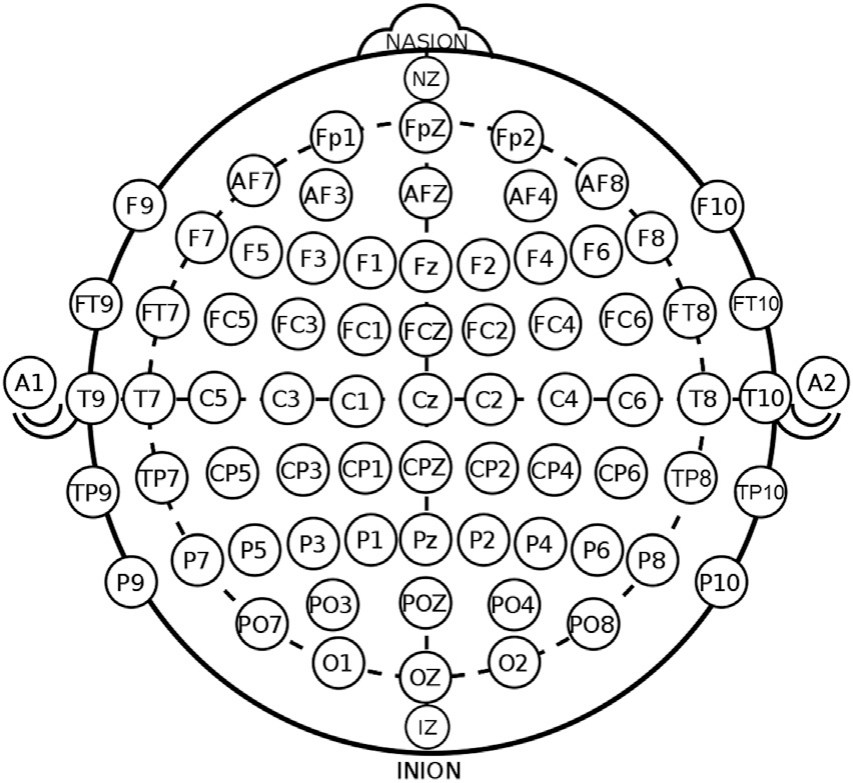
International 10-20 System for EEG-MCN by Oxley, B.C. (2020)^2^.

A point of theoretical departure in the present analysis—in relation to HTR—originates from an important study, reported a decade ago (Dubal et al., 2011). It demonstrated that *early P1 wave* was enhanced on the ten most posterior parietal–occipital electrode sites for emotional compared to neutral facial expressions, irrespective of the face mode—human or machine. Figure 1 presents the International 10-20 System for EEG-MCN (Modified Combinatorial Nomenclature) (Oxley, 2020). The main sections of the EEG array, denoting the respective brain areas, are given by capital letters as follows: F for frontal, P for parietal, O for occipital, T for temporal, and C for central. The odd numbers denote electrode placements on the left and the even numbers on the right; NASION is the frontal midpoint between the eyes, and INION is the end point at the back of the head (Schleiger et al., 2014).[Fn fn3]

According to the study by Dubal et al. (2011), the human brain detects the facial expression first, determines whether it expresses an emotion or neutrality, and only after that decides whether the face belongs to a human or a machine. This finding reveals a radical new understanding of the mechanisms of learning by the “social brain” (Dunbar, 2003), where more significant—in an evolutionary sense—are the most recent, social mechanisms, than the earlier perceptual saliency–based detection mechanisms (Lee et al., 2002; Elsherif et al., 2017). Therefore, a significant conceptual assumption can be made in the present context that the cognitive system *prioritises* information along with the ability to discriminate levels of *abstraction*, where the more abstract items are being processed earlier than the more concrete ones.

**FIGURE 2:**
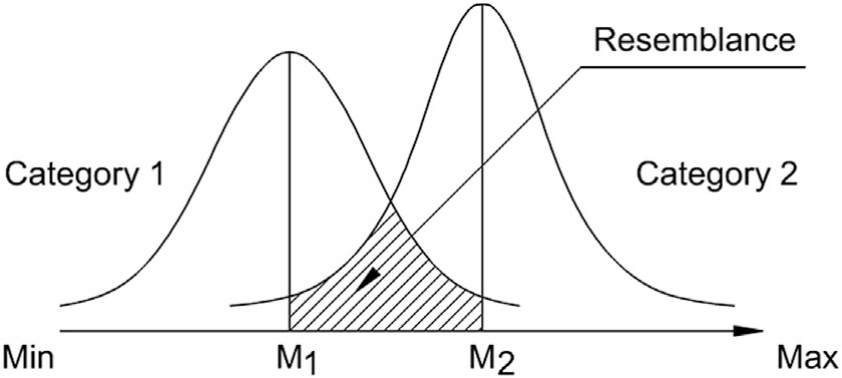
Conceptualisation of the process of subjective comparison of two categories.

This is in line with the categorical discrimination theory of Eleanor Rosch (Rosch and Mervis, 1975; Rosh, 1987). According to the theory, categories are being compared and subsequently discriminated along probabilistic dimensions, where the most central elements are the closest to the “feature family”, whereas at the outskirts are the rare and less characteristic features of a category. Within a similar probabilistic framework, Moore (2012) explained the emergence of the uncanny valley phenomenon (Mori, 1970; MacDorman, 2005), when people are initially attracted, but then repulsed at the encounter of robots, or androids, sharing “realistic” features with the humans—soft, but cold skin, staring eyes, etc. The effect emerges at the intersection of the broad distribution of possible “non-human” features (Category 1), and the narrower distribution of possible “human” features (Category 2), yet dependent on the internal subjective threshold within the overlapping “non-human” part onto the “human” area. Figure 2 is a representation of the conceptualisation of the process of comparison of two categories.

It was shown that the so-called “perceptual magnet effect” is also present in understanding robot utterances by the human (e.g., Read and Belpaeme, 2016), which, again, favours the position toward endowing humanoid robots with abstract “teacher assistant” abilities. Evidently, the effect of the uncanny valley emerges at the “clash” of abstract human-like features and the presence of concrete features like “cold skin” (which should be avoided in robot design). Therefore, the human brain responds to the *abstraction* with higher priority than to the set of immediate/*concrete* perceptual features. This ability makes possible to learn that a penguin is a bird and a bat is a mammal without any internal confusion. Therefore, we believe that robots can resemble human teachers in their deep, abstract features, irrespective of the appearances. Moreover, what the Dubal et al. study had revealed, this holds on the emotional level too. A robot’s display of emotions is, therefore, equally well accepted by the human learner as is the emotional reaction of the human teacher.

Extensive reviews of the EEG marker of involuntary attention called Mismatch Negativity (MMN), which is typically elicited between 100 and 150 ms in the fronto-central areas of the brain after the onset of a typically auditory stimulus, which is somewhat surprising or different, are provided in Duncan et al. (2009) and Justo-Guillén et al. (2019). Most often, the implemented paradigm is “oddball”—a succession of identical sounds, interspersed with different sounds. This is a situation of *abstract* succession processing, rather than detection of *concrete* stimuli. The MMN is an important pointer to the ability of the cognitive system to detect *abstraction* as a novel stimulus, capturing attention and, possibly, enhancing its memorisation *via* the episodic memory function to integrate novelty with a context for subsequent, more elaborate processing (Tulving, 1986).

### N170—Response to Facial Expressions—The Role of Distinctiveness in Memorisation of Concrete Features

The other important finding of the Dubal et al. study is the elicited later and lower N170 component by the machine face than the human face on the 19 most parietal–occipital electrode sites. It validates the overall experimental paradigm, since N170 reflects the structural encoding in face processing (Carmel and Bentin, 2002). Therefore, although the human viewer is completely aware that the emotion is conveyed by a machine, the goal is achieved in terms of understanding and memorisation of the presented by the teacher knowledge without any internal conflict taking place. In this way a robot can efficiently perform as a skilled assistant to the teacher, well accepted and enjoyed by the learners in this role.

Support to this view is provided by a study of different methodology than the one presented here. The oxygenated (HbO) and deoxygenated (HbR) hemoglobin levels were measured with a continuous wave functional near-infrared spectroscopy (fNIRS) system (measuring two wavelengths 690 and 830 nm) in Jung et al. (2016). This indirect method is almost as popular as EEG and is measuring changes both in oxy- and deoxyhemoglobin concentration but only from regions near the cortical surface. Evoked potentials (EPs) detect the cortical response to a given stimulus with high temporal resolution. fNIRS localises changes in oxygen metabolism following the neural activation. This phenomenon is called neurovascular coupling, allowing parallelism in the interpretation of the observed brain effects by both methodologies (Chiarelli et al., 2017).

In the study by Jung et al. (2016), two groups of participants viewed human vs robotic faces—neurotypical and participants with ASC^3^. The monitored sites are in the vicinity of T5 in the left hemisphere and T6 in the right hemisphere for a total of 14 channels (within the International 10-20 System as a reference for probe placement). Recordings were averaged within the time frame of 3–10 s after the stimulus onset. The neurotypical viewers showed greater right hemisphere lateralisation than the group with ASC when viewing human faces. However, no difference between the groups was observed in the levels of activation when viewing robotic faces. There was no difference in hemispheric activation, too. The authors interpret the finding of identical brain response to the robotic (but not human) faces in both groups as holding pedagogical potential for children with ASC since the processing of robotic faces is not impaired.

### Novelty P3—Levels of Response to Novelty—Perceptual vs Semantic Novelty

Another important aspect of the present analysis—in relation to SED—concerns the definition of the concepts of *novelty* and *surprise*. For example, children with ASC dislike novelty, which poses obstacles to the teaching process, especially in inclusive classes (e.g., Vivant et al., 2018). On the other hand, the human cognitive system, in general, is attracted by *novelty*, and this is an important factor in learning (e.g., Mather, 2013).

In Quent et al. (2021), five types of novelty/surprise signals are outlined—item vs context novelty, item vs context surprise, and complete novelty, following the model of Henson and Gagnepain (2010). A model is being described of the interrelation between distributions of the subjective experience of novelty or surprise in terms of existence of prior memory and the current expectation. Item novelty is defined by encountering an item, not seen before. Context novelty is defined in a similar way. Item surprise is defined by observing a familiar item with novel features, and context surprise—by observing a familiar context with novel features. Complete surprise is when item and context are not seen before. However, the item novelty definition seems rather vague—people do not encounter novel items often—after the age of 3—except in specially designed experimental conditions. On the other hand, the context novelty is experienced mainly with respect to the item—a familiar item can exist in a variety of familiar, yet unsuitable contexts. At the same time, new elements of familiar items or contexts seem the most frequent *novelty* encounters. Yet, if all are accompanied by the experience of surprise—this would be very resource consuming on the part of the cognitive system, since surprise implies certain emotional engagement.

**FIGURE 3:**
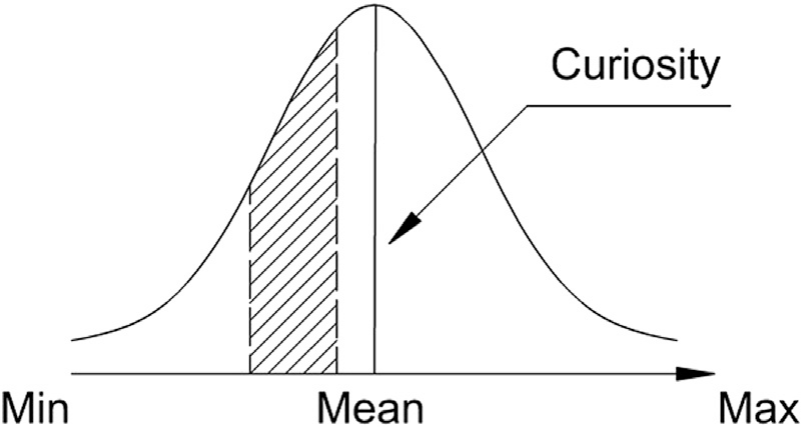
Area of the distribution of novelty effects in the brain, possibly attracting *curiosity* in learning new material of children with ASC.

It was shown in Reichardt et al. (2020) that *novelty* is often processed without being memorised. In order for a novel item or context to be remembered, some level of experiencing *surprise* should be present. In such a case, children with learning difficulties may experience additional issues with memorising items that they tend to avoid (as illustrated later in Figure 3).

**FIGURE 4:**
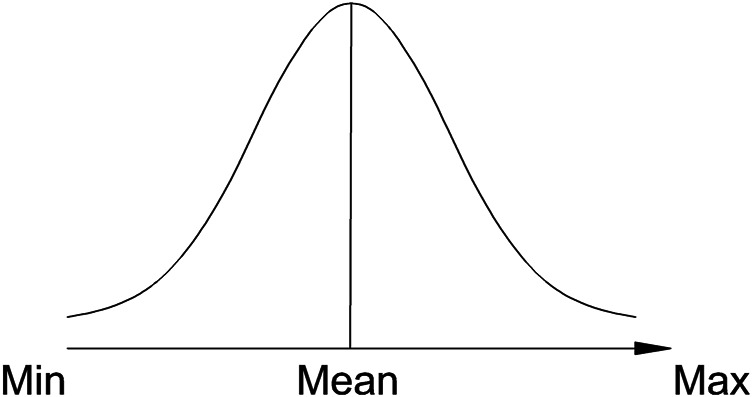
Representation of *novelty* as a subjective dimension from a minimum (Min) experienced novelty to a maximum (Max).

Our understanding of *novelty* is more straightforward. We assume *novelty* a dimension along its intensity—from min to max (Figure 4). We do not distinguish context from item, since these are inter-linked during learning, as Tulving has previously justified in numerous experiments (Tulving, 1983; Tulving, 1986; Tulving, 2002). Low novelty of the item-context configuration is defined by the lack of any noticeable new elements. High novelty is defined by a large proportion of novel elements in less familiar contexts.

The subjective experience of novelty is often explored within the “oddball” paradigm (e.g., Levi-Aharoni et al., 2020). The oddball paradigm is an experimental procedure where items (meaningful/meaningless; sensory/verbal; pictorial/spoken; etc.) are presented in a succession, and the EEG activation is observed in the range of 250–425 ms after the stimulus onset. In Barry et al. (2016), the oddball paradigm implements as stimuli nonverbal, auditorily presented sounds, bearing functional, rather than semantic content. The observed late subcomponent, following P3a and P3b, called Novelty P3, is said to be associated with the Orienting Reflex (e.g., MacDonald and Barry, 2020). It was observed at a peak latency of 335 ms. In the subsequent experiment, Novelty P3 was observed at a parietal site called parietal Novelty P3 (nP3p). A frontal Novelty P3 (nP3f) with a peak latency of 379 ms was also observed.

Within the oddball stimuli succession, the emergence of a *distinct* item invokes the Novelty P3 activation at the later stage of P3. The way the *distinct* item is being defined is an issue of ongoing debate. One way is to assume that it is a *novel item*, unprimed before (the most common case) (e.g., Nurdal et al., 2021). Another way is to define the *distinct* item as (*a priory*) *categorically different* from the rest, presented for the first time within the list, for example, a 3-letter syllable in a list of integers (e.g., Hunt, 1995). The better memorisation of the *odd* item gives support to the forwarded idea of the *abstract* level of cognitive processing in learning new material being given higher priority than the concrete level.

Studies show that the *distinctiveness* plays an essential role in the formation of episodic memories, with the substantial involvement of the hippocampus area of the brain (e.g., Hunt, 1995; MacLeod and Kampe, 1996; Dimitrova and Wagatsuma, 2011). However, *distinctiveness* is processed on different levels of *abstraction*—surface/perceptual/episodic memory based or deep/abstract/semantic memory based. The early activation components are possibly the familiar ones, whereas the late components represent the elaboration as a cognitive effort to integrate the novel item into contexts that become more and more familiar with the active involvement of the retrieval processes.

**FIGURE 5:**
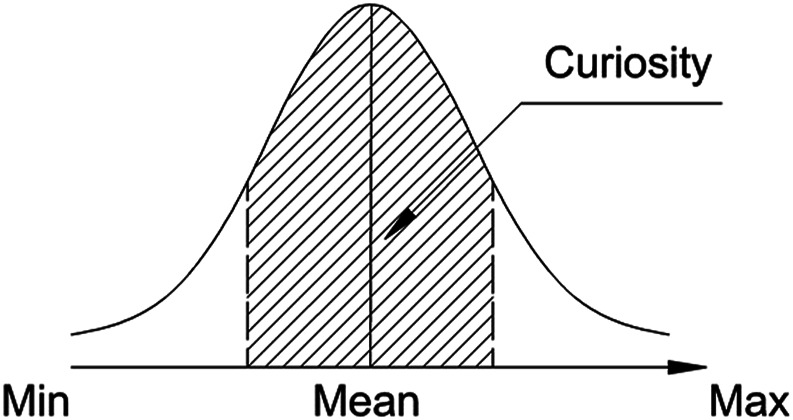
Area of the *novelty* distribution, attracting *curiosity* in learning new material.

The midrange of the distribution of novel elements is what drives *curiosity* in learning, which is often used as a basis for an intuitive teaching strategy by the educators (Figure 5). In Gruber and Ranganath (2019), it was argued that *curiosity* enhances the attention focus of the learner, involving increased dopaminergic activity, which can affect hippocampus-dependent consolidation of memory traces.

#### Semantic vs Perceptual Novelty—Cognitive Processing Priority of Abstraction Over Distinctiveness

By definition, the intensity of *surprise* is higher, than of *novelty*, due to the fact that in *novelty* no prior expectation is being formed as in *surprise*. In relation to surprise, Quent et al. (2021) reported observed involvement of the hippocampus combined with increased *motivation* and *exploration* (Kang et al., 2009; Gruber and Ranganath, 2019)—psychological processes being largely involved in classroom learning.

It is important to distinguish *semantic* from *perceptual* novelty. A perceptually novel stimulus is never seen before and will be observed as Novelty P3. A semantically novel stimulus, however, will have smaller amplitude on Novelty P3, since it is a novel feature of a familiar context, like in a folk story. Imagine a story with unusual or surprising elements. These often qualify as new items in familiar contexts. An example is a peach floating down the river in the folk story of Japan called Momotaro, or “peach boy”. When Momotaro meets a dog, a bird, etc., these animals produce sounds, unexpected and unfamiliar, which brings an effect on the part of the listener that is *perceptual* in nature (and their EEG manifestation will be explored in the future).

The *intensity* of the perceptual component in the experienced surprise is possibly bigger than the *intensity* of the semantic component. The reason is that no expectation has been formed during listening to the story for the forthcoming sounds, whereas the semantic processing relies on expectations how the story might develop—either correct or incorrect. When the expectation is incorrect—the person experiences some form of surprise (often negative as emotion). It is assumed that the elaboration, associated with processing of the perceptual *distinctiveness*, is higher than during semantic processing, so more cognitive resource will be involved and the learning will be felt as more effortful.

It was shown in a study implementing eye tracking that children with ASC fail to form correct expectations about the development of a video plot just before the emerging conflict, whereas neurotypical (NT) children successfully form correct anticipations about the video plot. The method of determining this is recording the blink rate, where blink suppression is observed just before the conflict (NT), or a little after it (ASC) (Shultz et al., 2011). Since the intensity of the experienced surprise is higher than that of the novelty, children with ASC are under stress more often, than the NT children. Being prone to sensory-perceptual stimulation, they meet difficulties in forming or retrieving *abstractions,* needed in order to be at advantage during learning (e.g., Vanpaemel and Bayer, 2021). This leads to the involuntary shift of the focus of curiosity toward the left side of the distribution of the subjective novelty dimension (Figure 3) in order to spare cognitive resource.

Such an interpretation of *abstraction* processing by children with ASC is in line with the previously forwarded by the authors of the present article “limited resource hypothesis” (Dimitrova et al., 2015). According to it, children with ASC involuntarily shift attention towards the automatic range, that is, are more susceptible to influences, eliciting *involuntary* attention and curiosity (the left side of the distribution of novelty effects in the brain, depicted in Figure 3). The right side of this distribution involves more elaborate mental processing of voluntary attention, driving curiosity (experienced as more effortful by all children).

The comparison of the striped areas in Figure 5 and Figure 3 in relation to the possible diagnostic ability of the cyber-physical system (CPS) in the present context is hypothetical. It is possible and feasible to further test this hypothesis by designing purely behavioural tests/games to involve children and assess their learning skills. The focus is on *curiosity—*whether the distribution of the expressed attraction to the task is symmetrical in a group of children*—*or skewed in one of the directions*—*the automatic processing range (involuntary attention), left, or the consciously controlled range (voluntary attention), right. The results in any case will provide valuable insights into the underlying neuro-cognitive processing of the human brain without involving any direct EEG measurement method.

### Learning Processes Revealed by Observing the EEG Activation Associated With Reward Processing—RewP (250–450 ms)

**FIGURE 6:**
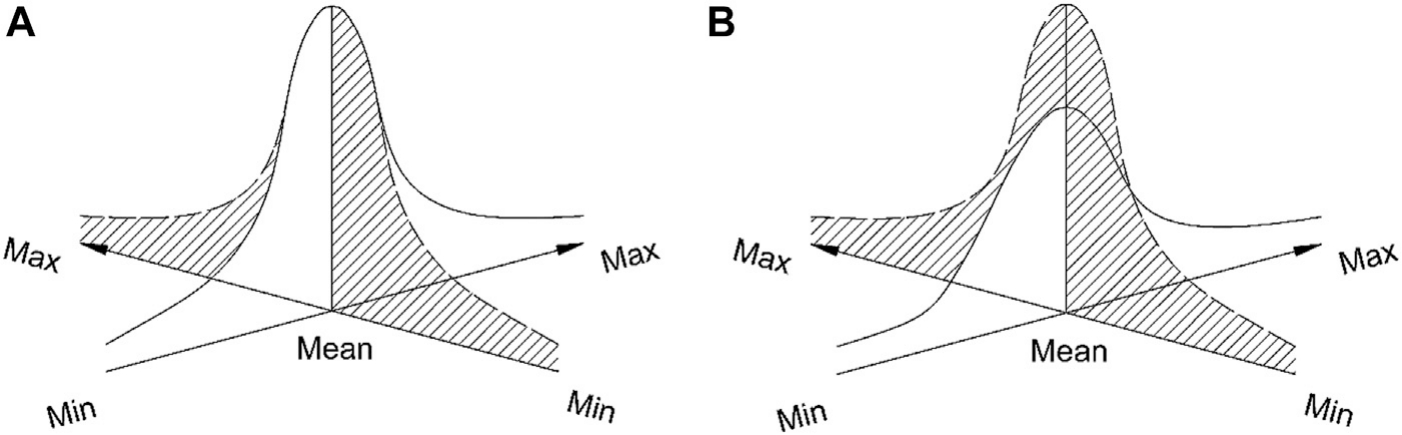
Visualisation of the possible intersection of the *novelty* processing (white) with the *reward* processing (striped) distributions in the subjective cognitive space in the case of similar standard deviations **(A)** and dissimilar standard deviations **(B)**.

The reward positivity (RewP) is a positive going fronto-central component, peaking around 250–450 ms after evaluative feedback of subject performance, being larger, when the reward is bigger (Brown et al., 2020). It is associated with mechanisms of the brain, acting as a predictive system, aiming to spare energy by constantly forming anticipations of future sensory stimulation, or events, as surveyed by Reichardt et al. (2020). One can imagine the experience when learning to drive a car—beginners experience *surprise* much more often than the experienced drivers, who are, nevertheless, often placed in situations of constant processing of *novelty*. Interestingly, *intrinsic motivations* (acting as a substitute of the reward in human learning) seem linked to novelty and surprise processing, rather than processing of unexpected or expected reward (Düzel et al., 2010; Caligiore et al., 2015). Moreover, Meng and Ma (2015) call the difference in the activation between success and failure outcomes of feedback-related negativity (250–350 ms) at the midline-frontal electrodes (d-FRN) “a real-time electrophysiological marker of intrinsic motivation (p. 445)”. Pornpattananangkul et al. (2019) for the first time defined EEG markers of motivation to play a game as different from the time for the choice to be made in the game (i.e., level of difficulty of the choice). Moreover, processing *motivation* preceded processing *how easy* was to make one choice over the other-around 300–600 ms at central-parietal sites, but motivation influences occurring around 200 ms earlier. This finding is yet another view supporting the multidimensional type of brain processing underlying learning and decision making. A future idea to be explored is the separate link to memorisation of processing novelty on the one hand and processing reward on the other. It is possible that the subjective dimensions of novelty and reward processing are orthogonal to each other as in Figure 6. Figure 6 presents a hypothetical visualisation of the possible intersection of the novelty processing with the reward processing distributions in the subjective cognitive space in the case of, possibly, similar standard deviations (A) or dissimilar standard deviations (B).

The intersection of the probability distributions represent the simultaneous effect of the underlying brain processing of *novelty* and *reward* along the theory of Torgerson (1958, p. 247). Therefore, it is promising to model the brain as a multidimensional processing system of various subjective states, like the association of processing reward and memorisation, vs novelty and memorisation, possibly with differential involvement of emotion. These aspects need to be explored in future studies.

It was also shown that processing novelty is related to *curiosity* as a state and a personality trait (Gruber and Ranganath, 2019). This novel finding demonstrates the feasibility to study brain processes of limited temporal scope (internal states) as separate from long-term, belonging to the personality of the learner. High curiosity as a trait requires a different way to approach the learner by the teacher than low curiosity. Yet accounting for the personality of the child is a factor to improve learning, especially in children with special learning needs.

The personality dimensions, theoretically, are infinite in number (e.g., Dimitrova, 2016). Two novel types of personalities were defined in a human-robot interaction context—socially motivated and socially indifferent (Dimitrova et al., 2019). Interestingly, the socially motivated learners in a zoology lesson, taught by a humanoid robot, gave reliably more positive comments on the robot itself, than the socially indifferent learners (contrary to any ad hoc expectation). This was interpreted as providing support to the socialising role of humanoid robots in class. The relation of social motivation, curiosity and the intrinsic motivations within a multidimensional brain processing system is still to be explored in future studies.

### The “Surprising” Role of Dispositions in Semantic Processing, and of Predictive Processing on the Uncanny Valley Subjective State—The Effects on N400

An EEG marker of semantic processes is a negative wave, the so-called N400 in response to words that cannot be integrated semantically into the sentence context, occurring at midline electrode sites (Fz, FCz, Cz, CPz, and Pz) (Dwivedi and Selvanayagam, 2021). Interestingly, people with negative personal disposition elicited attenuated N400. No correlation of the expression of positive disposition and the amplitude of N400 was observed. This finding is essential in focusing on children’s dispositions during learning, especially if the focus is on preserving cognitive resource. Further studies may bring interesting insights on the role of the dispositions on the learning outcome so that psychosocial and pedagogical rehabilitation^4^ approaches are also included in classroom work to make it more enjoyable in the future.

In Urgen et al. (2018), a robot (mechanical appearance), an android (human-like appearance), and a human performed everyday actions—drinking from a cup, waving hand, etc. The movements of the robot and the human were called *congruent*, since they were what could be expected—abrupt, robot-like for the robot and smooth, human-like for the human. The android, however, performed *incongruent* movements—since it moved abruptly (being mechanical), but looking like a human. EEG was recorded from human participants after a still, or a moving video of the stimulus. As expected by the authors, the N400 effect at the frontal sites (AF3, AFz, AF4, Fz, F1, F2, F3, F4, F5, and F6) of the moving android was the strongest in comparison to the rest, as well as when dynamic vs static conditions were compared. The explanation was that viewers formed *anticipation* of the behaviour of the observed agents—human, robotic, or android. The expected *congruence* is the strongest factor, which can be used as a guideline in the design of educational robotic systems for pedagogical rehabilitation in inclusive classes.

### Processing Abstraction at the Highest Level—Syntax and Semantic Effects on P600 and Detection of Mind Wandering

Syntactic processes are linked to a late centro-parietal positivity, termed P600, which occurs between 600 and 1,000 ms. The P600 correlates with processing of syntactically complex sentences (Daltrozzo and Conway, 2014). Delogu et al. (2019) claim that N400 is related to retrieval, rather than semantic processing, whereas P600 correlates with the difficulty to meaningfully integrate the sentence, especially when the sentence is syntactically correct but semantically improbable. According to Brouwer and Crocker (2017, p. 4) “… N400 amplitude is sensitive to the degree of semantic expectancy, while P600 amplitude is modulated by the nature of the comprehension task. It is the interplay between the systematic modulation of these latent components, due to their spatiotemporal overlap in the observed ERP signal, which explains the variance in WCS derived effects … ”, referring to the waveform component structure (WCF), underlying most of the mainstream analysis and explaining some observed inconsistencies. The abovementioned temporal overlap is consistent with the multidimensional view of brain processing, especially in a complex domain, such as simultaneous semantic and syntactic processing.

Table 1 presents summarisation of the EEG markers of abstraction, attention and memory in the present survey. The final column presents the respective pedagogical implications, substantiated by the discussed brain processes.

**TABLE 1:** Summarisation of the EEG markers of abstraction, attention, and memory in the present survey.

EEG marker (modality, ms after stimulus onset)	Brain location	Psychological effect	Pedagogical implication
P1 (positive, 100 ms)	10 most posterior parietal-occipital electrode sites Dubal et al. (2011)	Facial expression detection in human vs. machine faces	Abstract robotic features are accepted well by the viewer, thereby overcoming the uncanny valley effect (Moore, 2012)
Mismatch negativity (MMN) (negative, 100–150 ms)	Fronto-central areas of the brain Fz, Cz, C3, and C4 Duncan et al. (2009); Justo-Guillén et al. (2019)	Involuntary attention to surprising or different auditory stimulus	Detection of abstraction (in a lesson) as a novel stimulus, capturing attention and enhancing its memorisation via episodic memory Tulving. (1986)
N170 (negative, 170 ms)	19 most parietal-occipital electrode sites Dubal et al. (2011)	Later and lower N170 component elicited by the machine face than the human face, since N170 reflects the structural encoding of face processing Carmel and Bentin, (2002)	Successful discrimination of the human and the robot and understanding/memorisation of the presented lesson without internal conflict
Novelty P3 (positive, 250–425 ms)	Parietal site (nP3p) (peak latency at 335 ms), frontal (nP3f) (peak latency at 379 ms) MacDonald and Barry, (2020)	Detection of novelty and experiencing surprise; discrimination of perceptual vs. semantic novelty	Processes underlying curiosity in learning new knowledge; required balance of novelty-surprise according to the individual style of the learner
RewP (positive, 250–450 ms)	Fronto-central component Brown et al. (2020)	Forming anticipations of future sensory stimulation or events Reichardt et al. (2020). Intrinsic motivations (acting as a reward substitute in learning) seem linked to novelty and surprise processing, rather than to reward processing (Calligiore et al., 2015)	The brain acts as a multidimensional processing system of various subjective states. There is interplay of novelty and memorisation as well as reward and memorisation in learning
FRN, that is, feedback-related negativity (negative, 250–350 ms)	Midline-frontal electrodes Fz, FCz (Meng and Ma, 2015)	When participants could chose tasks, the difference in the activation (d-FRN) was big between success and failure outcomes. Meng and Ma (2015) call this d-FRN “a real-time electrophysiological marker of intrinsic motivation (p. 445)”	Intrinsic motivations of learners have to be determined in order to modulate the *novelty* in the lesson according to the learner style since *reward* and *internal motivation* seem to act as orthogonal factors in education (Figure 6)
N400 (negative, 400 ms)	Midline electrode sites (Fz, FCz, Cz, CPz, and Pz) Dwivedi and Selvanayagam, (2021)	The response to words that cannot be integrated in a sentence was attenuated in persons with negative disposition	Personality factors like disposition—positive or negative—influences the learning process, especially when semantic integration is the aim. This is a novel finding deserving further investigation
N400 (negative, 400 ms)	Frontal sites (AF3, AFz, AF4, Fz, F1, F2, F3, F4, F5, and F6) Urgen et al. (2018)	The effect was stronger for incongruent android movements than to congruent ones—human or robot	Behavioural congruence is an important factor in design of robotic technology for pedagogical rehabilitation
P600 (positive, 600–1,000 ms)	Central and posterior sites Delogu et al. (2019); Bornkessel-Schlesewsky and Schlesewsky (2008)	Reflects the difficulty to integrate a sentence, especially when syntactically correct, but semantically improbable	Comprehension of the learned material can be indirectly observed which can help design less effortful tests in class

In our future studies, we plan to use folk stories told by humanoid robots. The stories typically contain elements, requiring semantic integration, whereas syntactically these are consistent. An example is a boy jumps out of the peach, after it is cut in halves. These studies will enable more detailed investigation of processing of *abstract* information at the late event-related potential (ERP) activations during comprehension of a story and prediction of its later recall.

One perspective educational approach would be the detection of “mind wandering” during learning, which will help the teacher reduce the number of stressful examinations on the entire study material. Groot et al. (2021) report a study of combined fMRI-EEG and pupilometry on identifying markers of mind wandering in tasks, requiring attention on the part of the learner. It demonstrated the feasibility of 1) combined registration of neurological and behavioural (pupilomery) markers of subjective states and 2) possibility to obtain reliable markers of mind wandering in principle. For example, it was shown that pupil dilation during mind wandering is bigger than during the on-task attention. Attention deficit/hyperactivity disorder (commonly observed in children with ASC) was predicted by a set of EEG markers of spontaneous mind wandering in (Bozhilova et al., 2018). This type of studies is in its early stage, yet holding a large potential for future classroom situations.

## Conclusion

The article presented a survey of literature for a developed approach to implementing CPS, capable of diagnosing learner states so that the process of acquiring new knowledge becomes easier, more enjoyable and less effortful in future inclusive classes. The CPS will have to be endowed with an improved diagnostic ability for efficient technological support of the learner according to their individual style, pace, and interests.

In the present article, we focus mainly on EEG markers of *involuntary*, *implicit*, and *spontaneous* processing, since our aim is to identify strategies that capture learner attention and interest and avoid the effortful style of current education frameworks. We believe that the potential of the “amusing education” is extremely essential and capable of benefitting from the numerous present-day cognitive and neuro-cognitive studies.

A second specificity of the approach, presented in the article, is not focusing on the numerous studies, comparing the cognition of learners with ASC and of neurotypical students. The reason is the adopted approach to explore relevant knowledge from the general studies on brain processing in a variety of experimental paradigms. This type of knowledge is still largely unexplored in a systematic manner in relation to design of pedagogical scenarios and can be used to a greater extent in the design of novel pedagogical situations, including humanoid robots as valuable assistants of the teachers in class.

## Footnotes

1 Similar to the definition of “family resemblance” of Eleanor Rosch (Rosh and Mervis, 1975).

2 The file with the figure is made available under the Creative Commons CC0 1.0 Universal Public Domain Dedication.

3 We favour the approach, defining autism as a condition (ASC), but not disorder (ASD) (e.g., Cheng et al., 2010; Dimitrova, 2015).

4
https://cordis.europa.eu/project/id/777720
