# Corrigendum: A Review of Possible EEG Markers of Abstraction, Attentiveness and Memorisation in Cyber-Physical Systems for Special Education

**DOI:** 10.3389/frobt.2021.795160

**Published:** 2021-11-02

**Authors:** Maya Dimitrova, Hiroaki Wagatsuma, Aleksandar Krastev, Eleni Vrochidou, J. David Nunez-Gonzalez

**Affiliations:** ^1^ Department of Interactive Robotics and Control Systems, Institute of Robotics, Bulgarian Academy of Sciences, Sofia, Bulgaria; ^2^ Department of Human Intelligence Systems, Graduate School of Life Science and Systems Engineering, Kyushu Institute of Technology (KYUTECH), Kitakyushu, Japan; ^3^ Department of Computer Science, Human-Machines Interaction (HUMAIN) Lab, International Hellenic University (IHU), Kavala, Greece; ^4^ Department of Applied Mathematics, Engineering School of Gipuzkoa—Eibar Section, University of Basque Country (UPV/EHU), Bilbao, Spain

**Keywords:** EEG marker, abstraction, novelty, surprise, involuntary attention, memory, curiosity, cyber-physical systems for special education

In the original article, Moore (2012) was cited but the reference was missing. This reference has now been included in the References list.

The authors apologize for this error and state that this does not change the scientific conclusions of the article in any way. The original article has been updated.
